# Supplementary material for: Directed manipulation of membrane proteins by fluorescent magnetic nanoparticles
Source: Nat Commun. 2020 Aug 26;11:4259. doi: 10.1038/s41467-020-18087-3 (PMC7450064; doi:10.1038/s41467-020-18087-3)
Supplement: Supplementary file 1 — Supplementary Information [file 41467_2020_18087_MOESM1_ESM.pdf]

Supplementary Information

Directed manipulation of membrane proteins by fluorescent magnetic nanoparticles

Li et al.

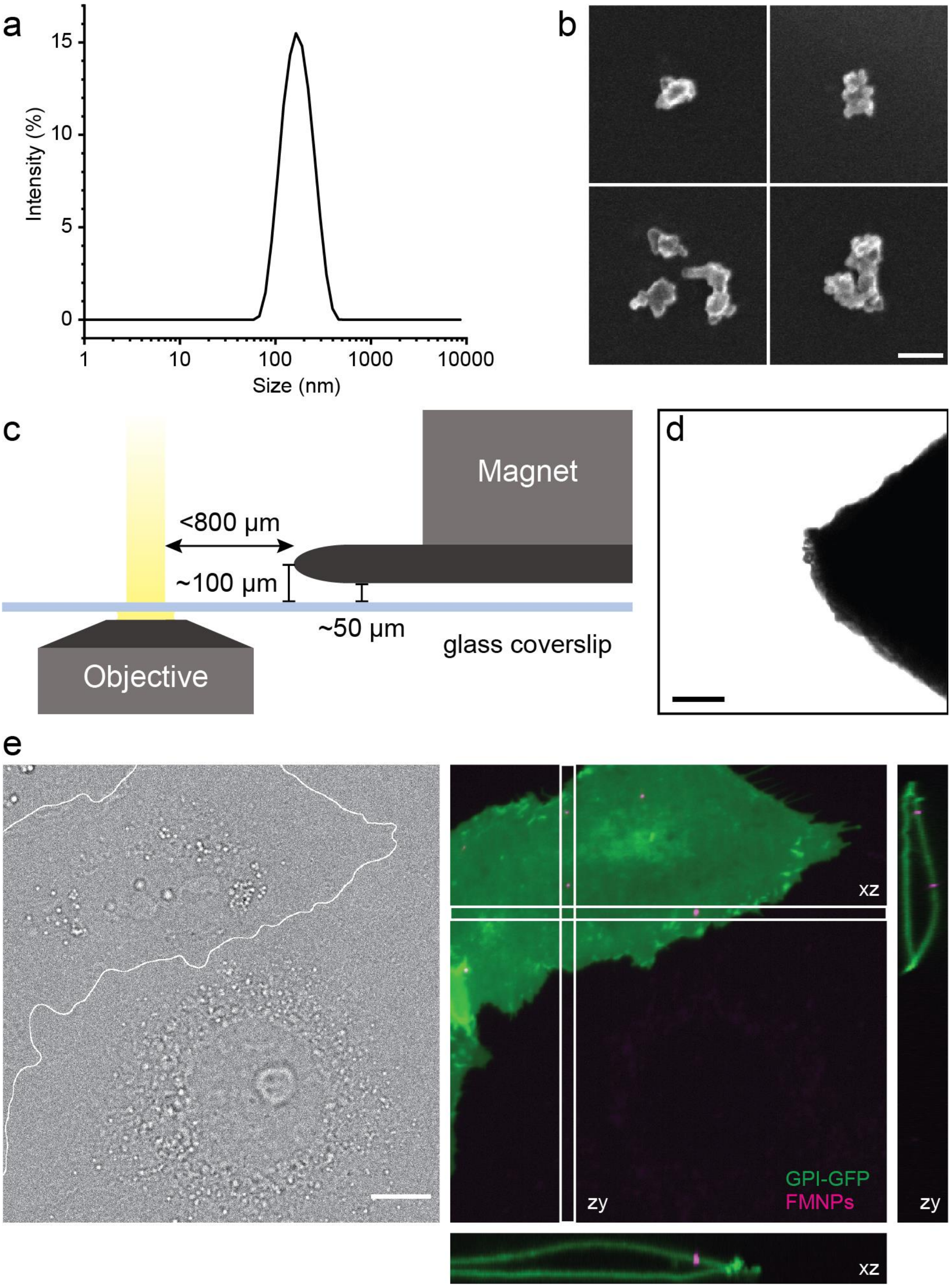

Supplementary Figure 1: Characterization of FMNPs, magnetic needle setup, and specific binding via nanobodies. a) Intensity-based particle size distribution from DLS measurements of FMNPs in PBS. Intensity averaged over four measurements against the particle size (hydrodynamic diameter). **b)** Scanning electron micrographs of representative single FMNPs. Scale bar is 100 nm. **c)** Scheme of the magnetic needle setup, not drawn to

scale. **d)** Transmission light micrograph of the tip of a representative needle. Scale bar is 10 μm. **e)** Left: GPI-GFP expressing (white outlined) and a wild-type CV-1 cell. Scale bar is 10 μm. Right: Average projection of a fluorescence z-stack shows that the nanobody-coated FMNPs (magenta) have bound specifically to GPI-GFP (green). Orthogonal views (zy and xz) indicate binding to the dorsal side of the cell.

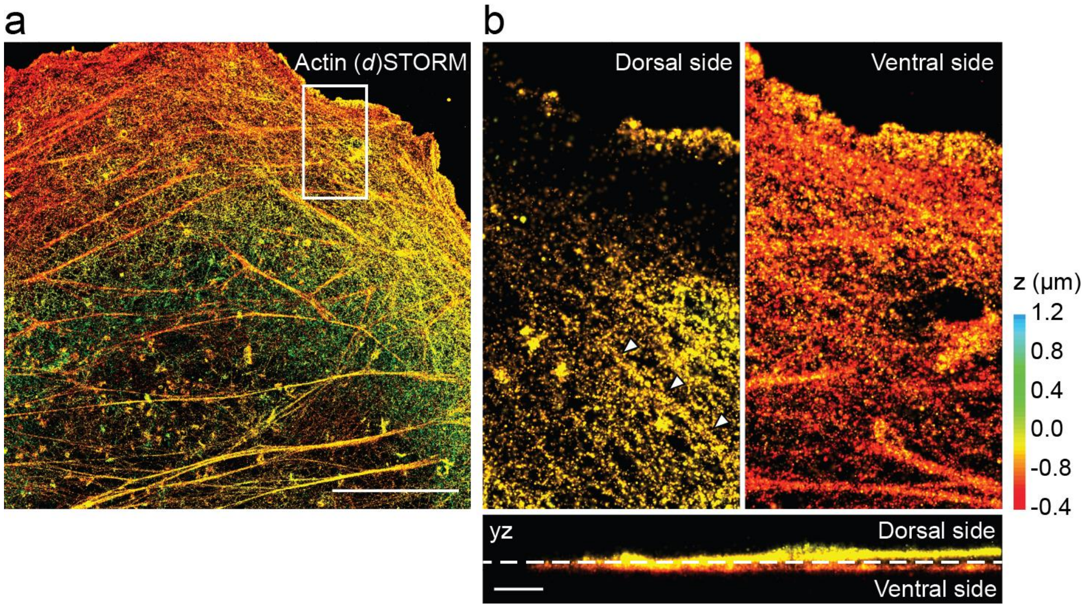

Supplementary Figure 2: The actin filament shown in Fig. 4 is located at the dorsal side of the plasma membrane. a) Full z-range reconstructed (*d*)STORM image of F-actin of the same cell as in Fig.4. Scale bar is 10 μm. **b**) Close-up view of selected z-slices of the dorsal (left) and ventral (right) cortical F-actin, as indicated in the yz view below. Scale bar is 1 μm.
