# Supplementary material for: Directed manipulation of membrane proteins by fluorescent magnetic nanoparticles
Source: Nat Commun. 2020 Aug 26;11:4259. doi: 10.1038/s41467-020-18087-3 (PMC7450064; doi:10.1038/s41467-020-18087-3)
Supplement: Supplementary file 3 — Description of Additional Supplementary Files [file 41467_2020_18087_MOESM3_ESM.pdf]

Description of Additional Supplementary Files

File Name: Supplementary Movie 1

Description: Reversible magnetic manipulation of a FMNP-bound lipid in a supported lipid bilayer. The same particle as in Fig. 1a (white arrow) bound to DSPE-PEG(2k)- biotin in a SLB was tracked before, during, and after magnetic manipulation. The magnetic tip was placed to the right side of the field of view. Single-particle trajectories are colored by time and overlaid onto the raw TIRF video. Playback speed is 5-fold of the real time speed.

File Name: Supplementary Movie 2

Description: A single FMNP bound to GPI-GFP on a live cell became immobile under magnetic manipulation. The particle moved to the right side of the field of view where the magnetic tip was placed, but then slowed down and finally became immobile. The movement is correlated with the cortical F-actin cytoskeleton as shown in Fig. 4. Single-particle trajectory colored by time and overlaid onto the raw fluorescence time-lapse video. Playback speed is 10-fold of the real time speed.
