# Supplementary material for: Directed manipulation of membrane proteins by fluorescent magnetic nanoparticles
Source: Nat Commun. 2020 Aug 26;11:4259. doi: 10.1038/s41467-020-18087-3 (PMC7450064; doi:10.1038/s41467-020-18087-3)
Supplement: Supplementary file 6 — Reporting Summary [file 41467_2020_18087_MOESM6_ESM.pdf]

nature research

Corresponding author(s): Helge Ewers

Last updated by author(s): Jul 28, 2020

Reporting Summary

Nature Research wishes to improve the reproducibility of the work that we publish. This form provides structure for consistency and transparency in reporting. For further information on Nature Research policies, see Authors & Referees and the Editorial Policy Checklist.

Statistics

For all statistical analyses, confirm that the following items are present in the figure legend, table legend, main text, or Methods section.

n/a	Confirmed	
☒	☐	The exact sample size (*n*) for each experimental group/condition, given as a discrete number and unit of measurement
☐	☒	A statement on whether measurements were taken from distinct samples or whether the same sample was measured repeatedly
☐	☒	The statistical test(s) used AND whether they are one- or two-sided<br>*Only common tests should be described solely by name; describe more complex techniques in the Methods section.*
☒	☐	A description of all covariates tested
☐	☒	A description of any assumptions or corrections, such as tests of normality and adjustment for multiple comparisons
☐	☒	A full description of the statistical parameters including central tendency (e.g. means) or other basic estimates (e.g. regression coefficient) AND variation (e.g. standard deviation) or associated estimates of uncertainty (e.g. confidence intervals)
☐	☒	For null hypothesis testing, the test statistic (e.g. *F*, *t*, *r*) with confidence intervals, effect sizes, degrees of freedom and *P* value noted<br>*Give P values as exact values whenever suitable.*
☒	☐	For Bayesian analysis, information on the choice of priors and Markov chain Monte Carlo settings
☒	☐	For hierarchical and complex designs, identification of the appropriate level for tests and full reporting of outcomes
☒	☐	Estimates of effect sizes (e.g. Cohen's *d*, Pearson's *r*), indicating how they were calculated

Our web collection on statistics for biologists contains articles on many of the points above.

Software and code

Policy information about availability of computer code

Data collection	Vutara SRX 6.04.14, Metamorph 7.8.6.0, NIS-Elements 6.0
Data analysis	Vutara SRX 6.04.14, Fiji/ImageJ 2.0.0-rc-69/1.52p; Java 1.8 0_66, Mosaic Suite 1.0.18, TrackMate 5.2.0, custom codes for Matlab 2018b: force calculation, track registration, D and track length filter

For manuscripts utilizing custom algorithms or software that are central to the research but not yet described in published literature, software must be made available to editors/reviewers. We strongly encourage code deposition in a community repository (e.g. GitHub). See the Nature Research guidelines for submitting code & software for further information.

Data

Policy information about availability of data

All manuscripts must include a data availability statement. This statement should provide the following information, where applicable:

- Accession codes, unique identifiers, or web links for publicly available datasets
- A list of figures that have associated raw data
- A description of any restrictions on data availability

The data that support the findings of this study are available from the corresponding author upon reasonable request.

Field-specific reporting

Please select the one below that is the best fit for your research. If you are not sure, read the appropriate sections before making your selection.

☒ Life sciences ☐ Behavioural & social sciences ☐ Ecological, evolutionary & environmental sciences

Life sciences study design

All studies must disclose on these points even when the disclosure is negative.

Sample size	Fig. 1d shows data on one SLB which was imaged over 20 min. Observations similar to those shown in Fig. 2 were made on >9 individual cells from at least 2 independent experiments per construct.
Data exclusions	Trajectories shorter than 50 frames were excluded because the calculated D is more reliable for longer trajectories. Trajectories of immobile particles ($D < 0.005\ \mu m^2/s$) were excluded as well. For the force calculation, the cutoff D was $0.01\ \mu m^2/s$, track length 200 and displacement 10 µm to exclude less mobile particles.
Replication	The force calibration experiments were repeated with similar results (3 independent experiments). The magnetic manipulation of GPI-GFP was repeated >10 times, >3 times for YFP-GT46 and twice for TfR-GFP. The magnetic manipulation of GPI-GFP and post-hoc (d)STORM imaging of actin was successfully repeated in 3 independent experiments with similar movement pattern of mobile particles moving around or transiently stopping at actin filaments. Complete immobilization during the recording time of a mobile particle at an actin filament as shown in Fig. 4 was observed only once.
Randomization	Randomization was not applicable.
Blinding	Blinding was not applicable.

Reporting for specific materials, systems and methods

We require information from authors about some types of materials, experimental systems and methods used in many studies. Here, indicate whether each material, system or method listed is relevant to your study. If you are not sure if a list item applies to your research, read the appropriate section before selecting a response.

Materials & experimental systems

n/a	Involved in the study	
☐	☒	Antibodies
☐	☒	Eukaryotic cell lines
☒	☐	Palaeontology
☒	☐	Animals and other organisms
☒	☐	Human research participants
☒	☐	Clinical data

Methods

n/a	Involved in the study	
☒	☐	ChIP-seq
☒	☐	Flow cytometry
☒	☐	MRI-based neuroimaging

Antibodies

Antibodies used	LaG-16 anti-GFP nanobodies from Fridy et al. were recombinantly produced in house
Validation	Validated on CV-1 cells expressing surface GFP or wild type, see Supplementary Figure 1

Eukaryotic cell lines

Policy information about cell lines

Cell line source(s)	CV-1 cells were a kind gift from the Helenius laboratory
Authentication	Cell lines were not authenticated
Mycoplasma contamination	Cells were tested negative for mycoplasma (monthly testing)
Commonly misidentified lines (See ICLAC register)	Not used
