# Supplementary material for: Directed manipulation of membrane proteins by fluorescent magnetic nanoparticles
Source: Nat Commun. 2020 Aug 26;11:4259. doi: 10.1038/s41467-020-18087-3 (PMC7450064; doi:10.1038/s41467-020-18087-3)
Supplement: Supplementary file 2 — Peer Review File [file 41467_2020_18087_MOESM2_ESM.docx]

Peer Review Information

**Journal:** Nature Communications

**Manuscript title:** Directed manipulation of membrane proteins by fluorescent magnetic nanoparticles

**Corresponding author name(s):** Dr Helge Ewers

**Editorial notes:**

**Transferred manuscripts**

This manuscript has been previously reviewed at another journal that is not operating a transparent peer review scheme. This document only contains reviewer comments and rebuttal letters for versions considered at *Nature Communications*.

**none**.

**Reviewer comments & decisions:**

**Reviewer comments, first version:**

Reviewer #1 (Remarks to the Author):

Li et al report the use of fluorescent magnetic nanoparticles (FMNPs) to manipulate lipid membranes while also enabling single-particle tracking at the same time. The work is overall interesting and could be useful to the field. I recommend publication in Nature Communications, and hope the below comments would be helpful for their revision:

1. The authors just mentioned “we found these particles to be monodisperse” and stated some numbers from dynamic light scattering. It would be helpful if scanning electron microscopy could be used to directly visualize the particles for better discussion of size.

2. The magnetic needle used to drag the FMNPs is critical in this study. It would be helpful to show some micrographs of the magnetic needle, and also better describe the geometry in this work. One thing I noticed from the Methods section is that the tip of the needle was “100 um above the focus of the glass surface”. If that is the case, how well could the needle control FMNPs? How far was the lateral distance from the tip of the needle to the FMNPs? Is it assumed that all FMNPs in the field of view experience the same force?

3. Fig. 1d, the x-axis is directly labeled as “F in x”. This might cause confusion, especially that the distribution necessarily shows negative values. It might be helpful to show “apparent F in x” as a secondary x axis on the top, and show the primary x axis at the bottom directly as the actually measured “apparent vx”.

4. Fig. 3b: it may be useful to overlay FP in the membrane with FMNP in color? Like Suppl. Fig. 1b?

5. Supplement movies: it would be useful to add in timestamp in each frame.


Reviewer #2 (Remarks to the Author):

Recommendation: Reject

This paper describes single particle tracking of fluorescent magnetic nanoparticles (FMNP) under force application in a model lipid and cell membrane. FMNPs were synthesized by many other labs and have used for biological application. The concept using magnetic force along with optical imaging including single particle tracking (e.g., magnetic polyplex) have been widely used previously. The authors applied this concept for GPI-anchored proteins to monitor the protein diffusion under force, investigating the cytoskeletal drag effects. However, it is unclear why FMNP provides better tracking capacity compared with previous magnetic force microscopy. Accordingly, the paper did not find any novel insight on GPI protein diffusion, which further diminishes the enthusiasm on this paper. It has been very well-known that GPI anchored proteins exhibited transiently confined diffusion due to the picket-fence effects by actin fibers. As described in the paper, traditional single particle tracking methods using gold particles or fluorescence beads can provide actin-mediated confined diffusion of membrane proteins with even higher temporal (microsecond) and spatial resolutions. Determination of more biophysically relevant information that was not possible with traditional methods (e.g. the force required for protein to escape from the cytoskeletal trap) would justify the use of this technique. My suggestion is that current manuscript does not meet the high standard novelty to be publishable in Nature Communications.

Other concerns:
1. Magnetic force applied on a single particle is a product of the magnetic moment and field gradient. Magnetic particles experiencing the magnetic field gradient is a function of distance between the particle and the external magnetic needle. The force defined in Figure 1d was rather constant and neglected this effect.

2. The relatively large size of FMNP compared to the transmembrane domain (for GPI anchored protein, it is a single lipid) would significantly perturb the diffusion dynamics of the target. Particle diffusion is inversely proportional to the size of particles. Even with the consideration of 50-100 fold higher viscosity of lipid membrane than water, the size of FMNP would significantly alter the dynamics.
Accordingly, the diffusion coefficient of a biotinylated lipid molecules was about 10 times slower than the value measured by other methods. Particularly, this would pose even more serious problems in measuring GPI-anchored protein dynamics, where the cell membrane is crowded by other membrane proteins and sugar residues. Nonspecific interaction of FMNPs with other proteins through van der Waals force, steric repulsion, charge interactions, and double layer interactions would also perturb the diffusion dynamics of the target proteins.

3. FMNPs are coated with several streptavidin molecules, which may crosslink membrane proteins. This may stimulate nonspecific cell signaling such as recruitment of clathrin pits, actin filaments, lipid rafts, and so on. Discussion on the crosslinking effects must be included.


Reviewer #3 (Remarks to the Author):

The manuscript entitled “Directed manipulation of membrane proteins by fluorescent magnetic particles” by Jai Hui Li details the use of a magnetic field produced by a needle probe to drag fluorescent magnetic particles attached to lipid-anchored or transmembrane across the plasma membrane of living cells at forces in the femtonewton range. This is combined with super-resolution microscopy of the actin cytoskeleton to correlate barriers to dragging to the actin cytoskeleton.

Overall, this is a well written manuscript that I believe would be of general interest to the biophysics community. They present an interesting technique and demonstrate the combination of this technique and super-resolution microscopy can directly show interaction of GPI-anchored proteins with the actin cytoskeleton.

Below I have some comments for the Authors.

1. Was the diffusion coefficient of the bead attached molecule (be it lipid-anchored or transmembrane) measured for free diffusion prior to and after dragging the particle. One might expect that beads that are linked to a single or small number of molecules could collect many more bound molecules during dragging which may show up as a shift in diffusion coefficient if the aggregation is large enough. Aggregation of molecules under the bead may be a problem as it might trigger other effects in live cells.

2. It is my understanding the magnetic needle is about 100 um above the artificial membrane or cells in these experiments. How far away is the needle in the lateral direction? Do the authors have an estimate of the force transverse to the membrane relative to the lateral dragging force?

3. You state that the calculated force was 9 +/- 11 fN over 469 tracks. This means a significant fraction of the particles dragged acted like they were experiencing a negative force and other looked like they experienced a nearly 4-fold larger force than the average. While I realize fluctuations are expected in these systems, how much of this variability could be due to the 3-D positioning accuracy of the needle relative to the cell considering the high field gradient near a needle point?

4. The section on dragging molecule-bead complexes in live cells does not give the diffusion coefficients (pre- and post-dragging) or dragging velocities under force. It would be useful to have these to compare with data already in the literature.

5. References 3, 11 and 24 are missing the volume and page numbers.

**Author rebuttal, first version:**

Reviewer #1 (Remarks to the Author):

Li et al report the use of fluorescent magnetic nanoparticles (FMNPs) to manipulate lipid membranes while also enabling single-particle tracking at the same time. The work is overall interesting and could be useful to the field. I recommend publication in Nature Communications, and hope the below comments would be helpful for their revision:

We thank the reviewer for this assessment of our work and hope that our improved manuscript is now acceptable.

1. The authors just mentioned “we found these particles to be monodisperse” and stated some numbers from dynamic light scattering. It would be helpful if scanning electron microscopy could be used to directly visualize the particles for better discussion of size.

We apologize for omitting electron microscopy images for easier visual access. We used DLS as a method of choice to measure particle size of colloidal materials and check for possible aggregation, since it gives an overview on the population level. We now document these data and include SEM images of FMNPs in Supplementary Figure 1.

2. The magnetic needle used to drag the FMNPs is critical in this study. It would be helpful to show some micrographs of the magnetic needle, and also better describe the geometry in this work. One thing I noticed from the Methods section is that the tip of the needle was “100 µm above the focus of the glass surface”. If that is the case, how well could the needle control FMNPs? How far was the lateral distance from the tip of the needle to the FMNPs? Is it assumed that all FMNPs in the field of view experience the same force?

We agree with the reviewer that we could document this in more detail. We now added a scheme of the magnetic needle setup and an image of the needle tip to Supplementary Figure 1. The 100 µm is a very conservative estimate for the very tip of the needle. We moved the needle as far down as possible on the coverslip surface without touching it. The needle is certainly closer than 100 µm. We do not have a direct comparison to other possible approaches, but we could pull hundreds of particles over the bilayer in every experiment this way and found the experimental procedure to be quite productive. Please find below a figure of all the trajectories imaged in one experiment. Scale bar is 10 µm.

Through refined analysis we could indeed visualize that the force experienced by a magnetic particle is distance-dependent (see new Figure 1). When we placed the needle adjacent to the field of view, the force increased with proximity to the tip within ~100 µm distance (Fig. 1d). When we placed the magnet several mm away from the sample, we could still detect directed motion and the velocity and calculated force seemed to decrease with larger distance, however at those distances the noise is higher relative to the displacement effect (plotted above), generating a large noise in the extraction of the magnetic force. The lateral distance from the tip to the field of view was between 0 and ~750 µm for different experiments shown (Supplementary Figure 1).

3. Fig. 1d, the x-axis is directly labeled as “F in x”. This might cause confusion, especially that the distribution necessarily shows negative values. It might be helpful to show “apparent F in x” as a secondary x axis on the top, and show the primary x axis at the bottom directly as the actually measured “apparent vx”.

We thank the reviewer for pointing out this cause of confusion and changed the figure. The distribution of the apparent vx is not identical to the distribution of the apparent Fx, although very similar, therefore we did not include the histogram of vx.

4. Fig. 3b: it may be useful to overlay FP in the membrane with FMNP in color? Like Suppl. Fig. 1b? We thank the reviewer for the suggestion and changed the Figure accordingly.

5. Supplement movies: it would be useful to add in timestamp in each frame.

We agree with the reviewer that this would make the data more accessible and changed the movies accordingly.

Reviewer #2 (Remarks to the Author):

This paper describes single particle tracking of fluorescent magnetic nanoparticles (FMNP) under force application in a model lipid and cell membrane. FMNPs were synthesized by many other labs and have used for biological application. The concept using magnetic force along with optical imaging including single particle tracking (e.g., magnetic polyplex) have been widely used previously.

The reviewer correctly states that magnetic nanoparticles have been used for biological applications and have been tracked with optical microscopy before. Indeed, we are indebted to fantastic pioneering work especially in the laboratories of Christoph Bräuchle, Armando E. del Río Hernández, Ellie Tzima, Peter A. Valberg, Young-Wook Jun, Thomas and Donna Jovin and the late Maxime Dahan (and others). However, we want to respectfully point out that the previously published work that inspired our experiments was mostly performed in the cytosol of cells and never reached the temporal (few ms) nor spatial (few nm) resolution of the work presented here. In the few studies where magnetic manipulation was applied to membrane proteins, quantification was lacking and the effect of magnetic pulling through the plasma membrane was not characterized in nearly as much detail and thus similar observations as we present could not be made. Of course, this does not reduce the merit of the pioneering abovementioned work, which we now discuss in greater detail in the manuscript.

The authors applied this concept for GPI-anchored proteins to monitor the protein diffusion under force, investigating the cytoskeletal drag effects. However, it is unclear why FMNP provides better tracking capacity compared with previous magnetic force microscopy. Accordingly, the paper did not find any novel insight on GPI protein diffusion, which further diminishes the enthusiasm on this paper. It has been very well-known that GPI anchored proteins exhibited transiently confined diffusion due to the picket-fence effects by actin fibers. As described in the paper, traditional single particle tracking methods using gold particles or fluorescence beads can provide actin-mediated confined diffusion of membrane proteins with even higher temporal (microsecond) and spatial resolutions. Determination of more biophysically relevant information that was not possible with traditional methods (e.g. the force required for protein to escape from the cytoskeletal trap) would justify the use of this technique.

Like the reviewer we are deeply interested in the interaction of membrane proteins with the cytoskeleton and this indeed is one of the motivations for the development of this approach. The reviewer is correct to point out that ms and µs tracking has found GPI-anchored molecules to be sensitive in their lateral motion to pharmacological disruption of actin filaments, has shown patterns that agree with actin mesh size and localizations could be correlated to super-resolved actin corrals. We however believe that the physical pulling of a protein through a membrane to sense the physical location of a barrier provides a new quality of direct evidence and an important novel approach for the investigation of the important regulation of membrane protein motion by the submembrane cytoskeleton. We are fully convinced that in the future our assay will open new doors to investigating membrane protein-cytoskeleton interaction.

My suggestion is that current manuscript does not meet the high standard novelty to be publishable in Nature Communications.

Other concerns:

1. Magnetic force applied on a single particle is a product of the magnetic moment and field gradient. Magnetic particles experiencing the magnetic field gradient is a function of distance between the particle and the external magnetic needle. The force defined in Figure 1d was rather constant and neglected this effect.

In previous Fig. 1d, we showed the global distribution of forces that particles experienced. We did not calculate the theoretical field force, but rather extracted the specific force exhibited from the movement of the particles. We have now refined our analysis and can indeed demonstrate a distance-dependent effect on the extracted forces within ~100 µm distance of the magnetic tip (Fig. 1d).

2. The relatively large size of FMNP compared to the transmembrane domain (for GPI anchored protein, it is a single lipid) would significantly perturb the diffusion dynamics of the target. Particle diffusion is inversely proportional to the size of particles. Even with the consideration of 50-100 fold higher viscosity of lipid membrane than water, the size of FMNP would significantly alter the dynamics. Accordingly, the diffusion coefficient of a biotinylated lipid molecules was about 10 times slower than the value measured by other methods. Particularly, this would pose even more serious problems in measuring GPI-anchored protein dynamics, where the cell membrane is crowded by other membrane proteins and sugar residues. Nonspecific interaction of FMNPs with other proteins through van der Waals force, steric repulsion, charge interactions, and double layer interactions would also perturb the diffusion dynamics of the target proteins.

We thank the reviewer for their concern regarding the accuracy of our SPT measurements. We do not claim that we provide the most accurate diffusion measurement possible. Like the reviewer, we are aware that such discussions are as old as the single particle tracking technique itself. As the reviewer pointed out, the viscosity of the plasma membrane of a cell is typically two orders of magnitude higher than that of culture medium. In a classical control experiment in Ken Jacobsons laboratory, 30% glycerol was added to the medium in an SPT experiment to determine if the viscous drag of the medium on the bead had any effect on lateral viscosity of a bound lipid in the plasma membrane. Such an effect was not detectable^1^. In this and other work^2^ the diffusion of paucivalent versus multivalent particles was investigated in a dilution chain of antibodies against Thy-1, a GPI-anchored protein attached to the beads. The effect was found to be small if detectable. While we are cautious in interpreting these data as the higher spatial and temporal resolution today should allow for the detection of smaller effects, we consider the size to be of minor influence, especially when comparing movement behavior with and without the magnetic needle. Furthermore, we can mention here that we used a nanobody with a PEG linker of ~10 nm length for linkage to the membrane proteins to evade possible nonspecific steric interactions of the nanoparticles with membrane molecules.

3. FMNPs are coated with several streptavidin molecules, which may crosslink membrane proteins. This may stimulate nonspecific cell signaling such as recruitment of clathrin pits, actin filaments, lipid rafts, and so on. Discussion on the crosslinking effects must be included.

We are aware that potential crosslinking is a general problem for the use of nanoparticles which has also been raised for other commonly used particles like gold nanoparticles and quantum dots. We agree with the reviewer that some crosslinking may take place here. The FMNPs are indeed coated with ~30 Streptavidin molecules per particle on average. We employed different experimental strategies to tackle this problem. On the supported lipid bilayers, the streptavidin-coated FMNPs are added directly and may accumulate biotinylated lipids untill all binding-sites are saturated. Here, our strategy to avoid crosslinking was to dope the lipid bilayer with only a minute amount of biotinylated lipid (0.001 mol%). In contrast, on cells we used monovalent nanobodies as intermediary binder which allows us to tune the binding stoichiometry. We made sure to limit the number of nanobodies per FMNP by titrating the amount of nanobodies vs. FMNPs. For the final experiments, a molar ratio of 1:1 nanobody to particle renders most particles to contain only one binding site or none. Considering Poissonian distribution, particles with more than one nanobody will still be present but the nanobodies would need to be bound in a steric conformation which allows for productive multivalent binding. In conclusion, we made sure to mitigate this issue as far as possible but since we cannot exclude residual crosslinking, we now included a discussion of possible effects of crosslinking in the manuscript.

Reviewer #3 (Remarks to the Author):

The manuscript entitled “Directed manipulation of membrane proteins by fluorescent magnetic particles” by Jai Hui Li details the use of a magnetic field produced by a needle probe to drag fluorescent magnetic particles attached to lipid-anchored or transmembrane across the plasma membrane of living cells at forces in the femtonewton range. This is combined with super-resolution microscopy of the actin cytoskeleton to correlate barriers to dragging to the actin cytoskeleton.

Overall, this is a well written manuscript that I believe would be of general interest to the biophysics

community. They present an interesting technique and demonstrate the combination of this technique and super-resolution microscopy can directly show interaction of GPI-anchored proteins with the actin cytoskeleton.

We are glad to hear the reviewer shares our excitement for this technique and hope we could further improve the value of our work for the community.

Below I have some comments for the Authors.

1. Was the diffusion coefficient of the bead attached molecule (be it lipid-anchored or transmembrane) measured for free diffusion prior to and after dragging the particle. One might expect that beads that are linked to a single or small number of molecules could collect many more bound molecules during dragging which may show up as a shift in diffusion coefficient if the aggregation is large enough. Aggregation of molecules under the bead may be a problem as it might trigger other effects in live cells.

We thank the reviewer for raising this interesting point we had not thoroughly analyzed yet. On SLBs, we rarely observed a decrease in mobility over the course of the magnetic pulling. On the other hand, we also observed particles increasing in mobility while being pulled, but similarly rarely. In most cases the particles resumed Brownian motion with a similar diffusion coefficient after the magnet was removed. We do observe particles becoming immobile during imaging but irrespective of the application of the magnetic needle. In most cases the particle is released while still being under magnet pull, sometimes only after removal of the needle. We assume that this observation was due to the particle encountering an amorphous impurity in the membrane from which it can escape stochastically. We feel that the release of the particles from being stuck and the resumption of free diffusion with the previous D after the magnet was removed is strong evidence that the engagement of the magnet does not induce lasting effects on FMNP-membrane interaction.

On cells, the possibility of binding additional molecules during dragging is considerably reduced for two reasons: first, the use of monovalent nanobodies at a 1:1 molar ratio limits the fraction of multivalent FMNPs. Secondly, the number of binding partners on the cell surface is relatively high due to the overexpression and therefore it is likely that possible binding events have occurred before applying the magnetic drag (~20 - 50 min after start of incubation with FMNPs). The diffusion coefficient varies over the course of an experiment (see below, left, data for GPI-GFP from 3 cells, 2 examples each) and no significant difference can be detected pre- and post-pulling (see below, right, paired data of GPI-GFP from 10 cells of 3 experiments) While we cannot exclude that the acquisition of additional binding molecules may play a role, we would mainly attribute changes in the movement behavior to interactions with the underlying cytoskeleton or extracellular matrix.

2. It is my understanding the magnetic needle is about 100 um above the artificial membrane or cells in these experiments. How far away is the needle in the lateral direction? Do the authors have an estimate of the force transverse to the membrane relative to the lateral dragging force?

We have added a scheme of the magnetic needle setup in Supplementary Figure 1 for clarification. While we cannot determine the transverse force, the perpendicular component of the force vector has likely no practical effect as the force required to pull a lipid out of a membrane bilayer is ~ 50 pN^3^ which is several orders of magnitude larger than the magnetic forces generated in this study (see revised Figure 1d) and hence we did not observe any indications for membrane deformation. Furthermore, detachment of particles did not detectably correlate with engagement of the magnet.

3. You state that the calculated force was 9 +/- 11 fN over 469 tracks. This means a significant fraction of the particles dragged acted like they were experiencing a negative force and other looked like they experienced a nearly 4-fold larger force than the average. While I realize fluctuations are expected in these systems, how much of this variability could be due to the 3-D positioning accuracy of the needle relative to the cell considering the high field gradient near a needle point?

The variability of the calculated force in the presence of the magnetic needle is in the same range as the variability in the absence of the magnetic needle; therefore, it is mainly due to the inherent noise of random diffusion and variability in the saturation magnetization between individual particles. The force calibration was performed on a planar model membrane with consistent needle positioning. For experiments on cells, the needle cannot be placed in the same position in respect to the 3D topology of the cell surface. Therefore, the calculated forces rather estimate the order of magnitude of the force experienced by the membrane molecules.

4. The section on dragging molecule-bead complexes in live cells does not give the diffusion coefficients (pre- and post-dragging) or dragging velocities under force. It would be useful to have these to compare with data already in the literature.

We thank the reviewer for this suggestion and have now analyzed our trajectories accordingly. We now discuss these data in the manuscript text, specifically, we now write in the manuscript: “Freely diffusing particles exhibited no detectable change in diffusion coefficients when they resumed random motion after being dragged across the cell (0.046/0.027 µm^2^/s (mean/median) before, 0.040/0.027 µm^2^/s (mean/median) after magnetic dragging (n. s., Student’s t-test)).“

Unfortunately, we could not find any data on membrane protein dragging velocities under magnetic force in the literature. The dragging velocity we measured was 0.23 µm/s ± 0.18 µm/s (n = 9). We now state this in the manuscript text. Diffusion coefficients for GPI-GFP are at 0.051/0.038 μm^2^/s mean/median comparable to previously published results by us ^4^ and others ^5^. The diffusion coefficients we yielded from particles before and after pulling are in the same range (see also our answer to question 1).

5. References 3, 11 and 24 are missing the volume and page numbers.
   We apologize for the omission and changed the entries accordingly.

References:

1. Lee, G. M., Ishihara, A. & Jacobson, K. A. Direct observation of brownian motion of lipids in a membrane. *Proc. Natl. Acad. Sci.* **88**, 6274–6278 (1991).
2. Sheets, E. D., Lee, G. M., Simson, R. & Jacobson, K. Transient confinement of a glycosylphosphatidylinositol-anchored protein in the plasma membrane. *Biochemistry* **36**, 12449–12458 (1997).
3. Stetter, F. W. S., Cwiklik, L., Jungwirth, P. & Hugel, T. Single lipid extraction: The anchoring strength of cholesterol in liquid-ordered and liquid-disordered phases. *Biophys. J.* **107**, 1167– 1175 (2014).
4. Albrecht, D. et al., Nanoscopic compartmentalization of membrane protein motion at the axon initial segment. *J Cell. Biol.* **215** 37-46 (2016).
5. Umemura, Y. M. *et al.* Both MHC class II and its GPI-anchored form undergo hop diffusion as observed by single-molecule tracking. *Biophys. J.* **95**, 435–450 (2008).

**Reviewer comments, second version:**

Reviewer #1 (Remarks to the Author):

The authors have addressed all my questions and improved the manuscript accordingly. I recommend publication.


Reviewer #3 (Remarks to the Author):

I believe the Authors have addressed all of my comments to my satisfaction and have no further reservations for publication.

As a note, though, regarding my point on the transverse forces, I was not thinking the forces could extract the probe molecule but that even minuscule transverse forces might locally bend the membrane allowing it to "bubble up" (for lack of a better term) in between cytoskeletal pinning points. Since the bilayer is fluid, excess membrane could flow in between pinning points and drive the probe towards the highest mid-point between pinning sites and thus reduce any interactions with the underlying cytoskeleton. After the inclusion of the schematic of the needle's positioning, I am less concerned, but I think the Authors might keep it in mind that interactions may be reduced in some situations.

**Author rebuttal, second version:**

**Reviewer comments, third version:**

**Author rebuttal, third version:**
